# Navigating the Spectrum: Atypical Pulmonary Involvement in Immunoglobulin G4-Related Disease

**DOI:** 10.7759/cureus.50086

**Published:** 2023-12-06

**Authors:** Muhammad Riazuddin, Maha Ali, Dalal F Alageel, Mais W Gheith, Saad S Ali, Noha I Farouk, Belal N Sabbah, Aamir Nadeem M Ali Sheikh, Khaled Alkattan

**Affiliations:** 1 Medicine, King Faisal Specialist Hospital and Research Centre, Riyadh, SAU; 2 College of Medicine, Alfaisal University, Riyadh, SAU; 3 College of Medicine, Dar Al Uloom University, Riyadh, SAU; 4 Thoracic Surgery, College of Medicine, Alfaisal University, Riyadh, SAU

**Keywords:** immunoglobulin g-4 (igg4) related systemic disease, igg4 disease, immune disorder, igg4 pleural involvement, igg4 -related disease

## Abstract

Immunoglobulin G4-related disease (IgG4-RD) is a systemic condition known to affect multiple organ systems. While its manifestations are diverse, pulmonary involvement, especially of the pleura, remains less common. We report the case of a 99-year-old Saudi male with a medical history of diabetes mellitus, chronic kidney disease, and hypertension. He presented with dyspnea and syncope, with radiological findings revealing pleural effusion and a mass in the right hemidiaphragm. Laboratory investigations highlighted elevated serum IgG4 levels, and histopathological evaluation confirmed the diagnosis of IgG4-RD. Notably, the patient's thoracic histopathology differed from typical IgG4-RD presentations, emphasizing the variability of the disease. This case underscores the significance of recognizing IgG4-RD as a potential cause of unexplained pleural effusion. It also highlights the need for a comprehensive diagnostic approach, integrating laboratory values, histopathological findings, and clinical context. Given the potential variability in thoracic IgG4-RD histopathology, clinicians should maintain a heightened awareness of this condition to avoid missed diagnoses.

## Introduction

Immunoglobulin G4 (IgG4)-related disease (IgG4-RD) is a comprehensive immune-driven disorder that has garnered attention in the medical community due to its ability to induce fibroinflammatory changes across a multitude of organ systems. This systemic condition is not limited to any single organ but can manifest in various parts of the body [1], making its diagnosis and management a challenge for clinicians.

The hallmark features of IgG4-RD include heightened IgG4 serum concentrations, which often serve as a diagnostic clue. Furthermore, histopathological examinations of affected tissues frequently reveal fibrotic changes. These fibrotic areas are accompanied by pronounced lymphoplasmacytic infiltration, indicative of the body's immune response. A particularly distinguishing feature is the marked presence of IgG4-positive plasma cells, which are often used as a criterion for diagnosis [2].

Among the various organs impacted by IgG4-RD, the lungs stand out due to the unique diagnostic challenges they present. Lung lesions associated with this condition are typically identified via CT-guided biopsy [3]. A prospective cohort study has shed light on the prevalence of thoracic symptoms in IgG4-RD patients. Around 35.1% of these patients manifest diverse thoracic symptoms, ranging from mild discomfort to severe respiratory distress. Above all, it is significant to shed light on the prevalence of various manifestations on imaging, such as hilar and mediastinal lymphadenopathy (52.9%), solid nodular (25.3%), round-shaped ground glass opacities (9.2%), alveolar-interstitial type infiltrates (20.7%), pleural effusion (4.6%), pleural nodules or thickening (16.1%), and bronchovascular type (23%) [4].

A somewhat significant aspect of IgG4-RD's pulmonary manifestations is pleural involvement. While it might seem like a minor component, pleural involvement was observed in about 4.6% of IgG4-RD cases [4]. This statistic underscores the importance of comprehensive pulmonary evaluations in patients suspected of having IgG4-RD to ensure accurate diagnosis and timely intervention. In this case, we present a 99-year-old man with unexplained pleural effusion and an incidental right hemidiaphragm mass. This case presents a rare manifestation of IgG4-RD, a disease process whose diverse manifestations are still being studied due to the large variety of presentations.

## Case presentation

A 99-year-old Saudi male with a notable medical history of controlled diabetes mellitus, chronic kidney disease, and hypertension presented with recent episodes of dyspnea and syncope in July 2021. His past surgical and interventional history is significant for an endoscopic retrograde cholangiopancreatography (ERCP) and choledocholithotomy due to acute biliary pancreatitis and cholelithiasis, with subsequent stent placement followed by removal in 2015. Additionally, he underwent percutaneous coronary intervention (PCI) targeting the left anterior descending artery to the left circumflex coronary artery with stent placement in 2015 and a PCI to the left main coronary artery in 2017.

The patient's dyspnea had an onset two days prior to presentation and had been progressively worsening. It was accompanied by orthopnea and right-sided pleuritic chest pain. He also reported syncope episodes, which were associated with falls but without any loss of consciousness. No other neurological manifestations were observed.

Upon examination, vital signs were within normal limits. Notably, his trachea was deviated to the right. Palpation and auscultation of the right lower lobe revealed dullness, and breath sounds were absent in this region. The cardiovascular examination identified a systolic ejection murmur graded at 3/6 at the left sternal border. Examinations of the abdomen, nervous system, and skin did not yield any significant clinical findings.

Laboratory investigations highlighted an elevated WBC count, decreased hemoglobin levels, an increased number of nucleated RBCs, mild hyperkalemia, mild hyponatremia, elevated alkaline phosphatase, and elevated C-reactive protein (CRP).

**Figure 1:**
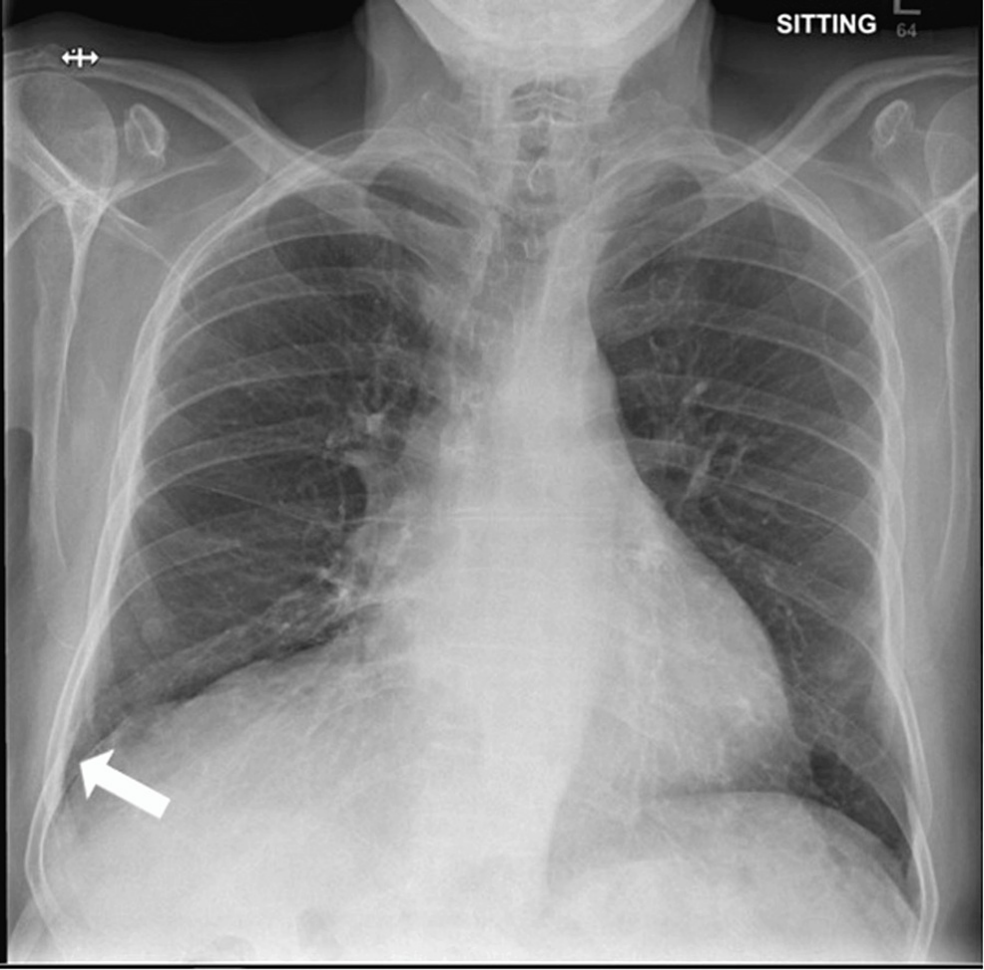
Chest X-ray from October 2020 showing right lower lobe consolidation or atelectasis and a small right-sided pleural effusion (white arrow). The left costophrenic angle appears clear, with no evident signs of pneumothorax.

**Figure 2:**
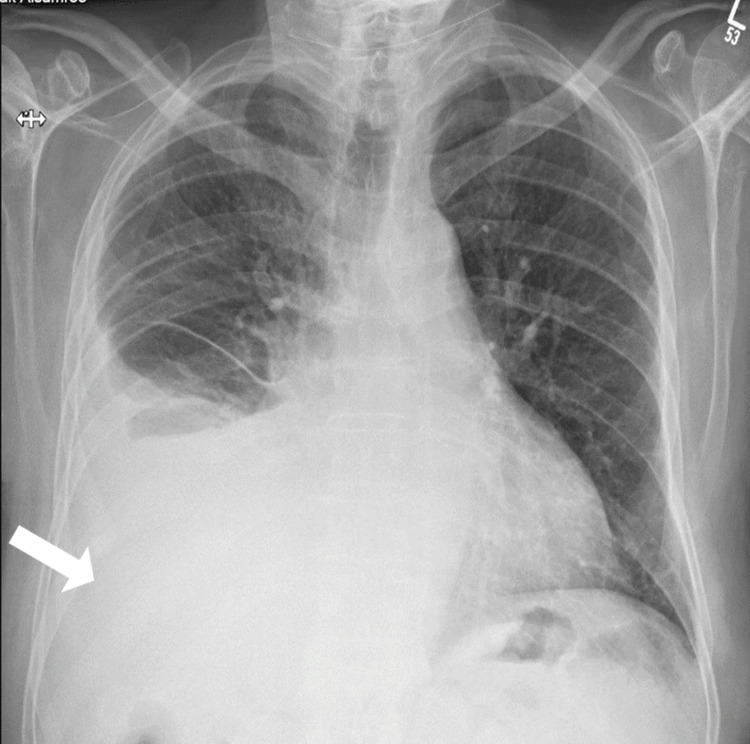
Chest X-ray from July 2021 illustrating the progression of the disease. The image reveals a small right-sided pleural effusion (white arrow) with adjacent collapse or consolidation. The left lung remains clear, and no signs of pneumothorax are visible.

Radiologically, a chest X-ray from October 2020 (Figure 1) demonstrated right lower lobe consolidation or atelectasis and a small right-sided pleural effusion. The left costophrenic angle was clear, and there were no indications of pneumothorax. A subsequent chest X-ray in July 2021 (Figure 2) showed progression of the disease with new findings, including a moderate right-sided pleural effusion with adjacent collapse or consolidation. The left lung appeared clear, and no pneumothorax was discernible.

**Figure 3:**
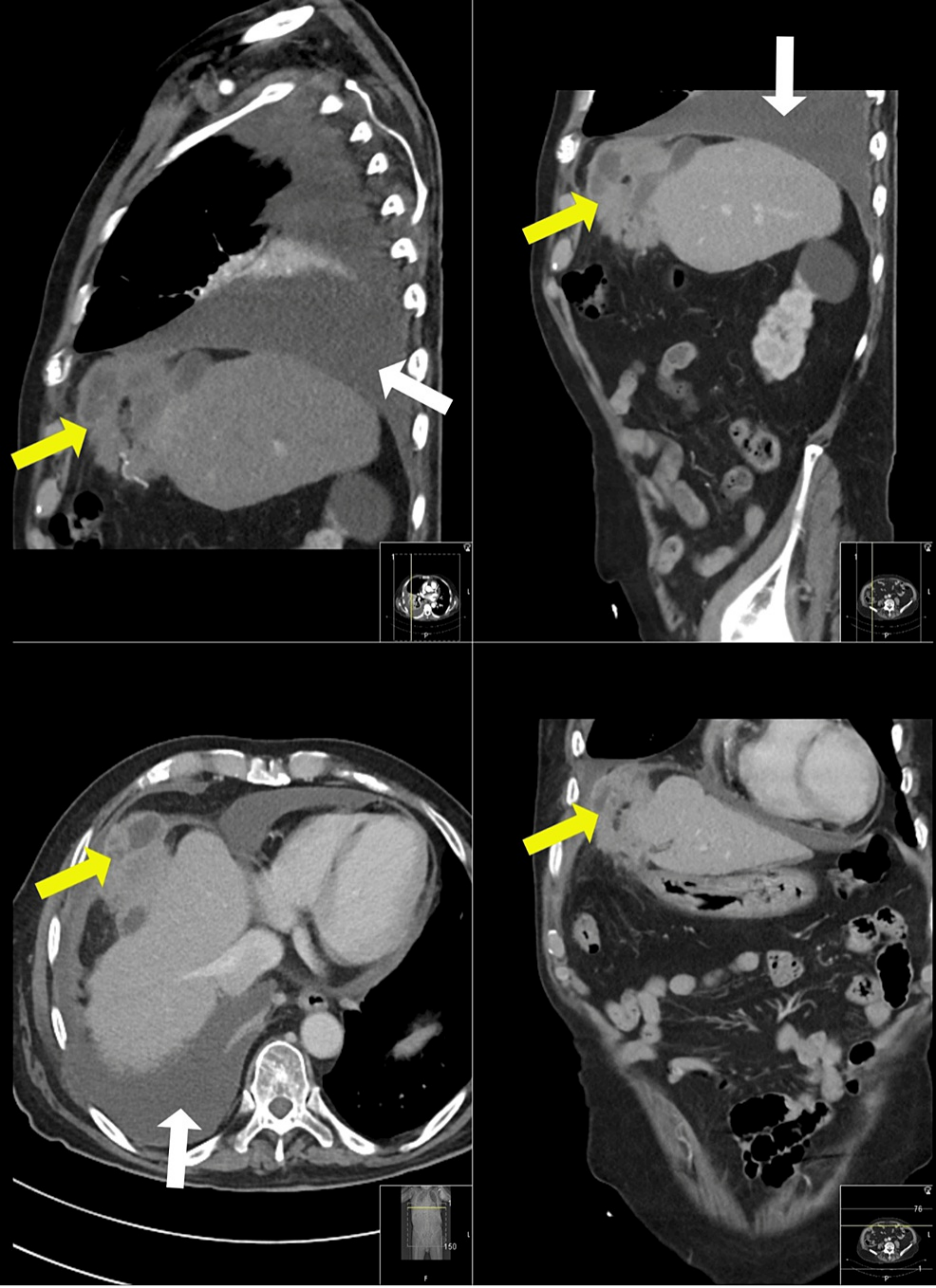
CT shows pericardial/pleural effusions, right lower lobe collapse (white arrows), and abnormal density at the right hemidiaphragm (yellow arrows).

To further evaluate the extent of the disease, a head CT without contrast was performed. Axial non-enhanced CT images of the head, complemented by sagittal and coronal reformations, did not reveal any acute intracranial abnormalities. A CT of the chest, abdomen, and pelvis (CT CAP) (Figure 3) indicated that the cardiac chambers and major mediastinal vessels appeared normal, except for severe coronary artery disease. A mild to moderate pericardial effusion was identified, along with several prominent mediastinal lymph nodes, the largest measuring 0.7 cm. The CT findings corroborated the chest X-ray observations, particularly concerning the right lung. This included a medium-sized pleural effusion leading to an almost complete collapse of the right lower lobe, compensatory hyperinflation of the upper and middle lobes, and the absence of pneumothorax. Additional findings included bilateral interlobular septal thickening, bronchial wall thickening, and dependent mosaic attenuation, which can be attributed to the pleural effusion. A significant observation was a heterogeneous soft tissue density (measuring 7.2 x 7.1 x 4 cm) located at the anterolateral aspect of the right hemidiaphragm, accompanied by reactive diaphragmatic thickening and minimal surrounding fat stranding.

Pleural fluid analysis yielded results of exudate with negative bacterial and mycobacterial cultures. Notably, adenosine deaminase was elevated. Cytology showed inflammatory cells (predominantly lymphocytes) with no evidence of malignancy.

**Figure 4:**
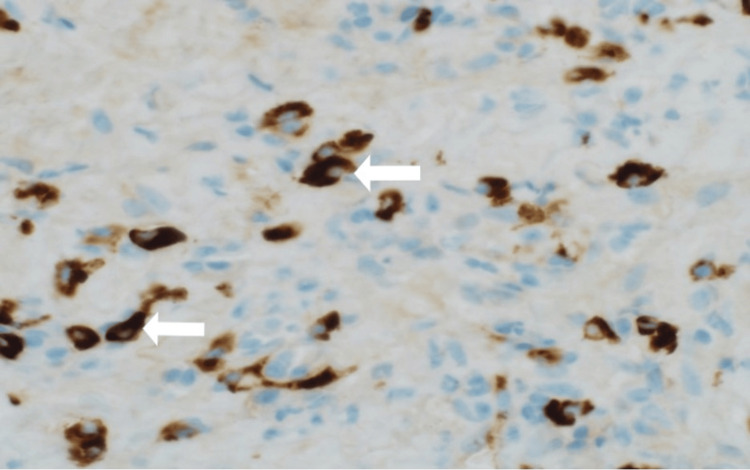
Immunohistochemical staining revealed IgG4-positive plasma cells.

A CT-guided biopsy of the right pleural mass was obtained, and the results showed xanthogranulomatous inflammation with immunohistochemistry revealing an increase in IgG4-positive plasma cells (Figure 4). The culture was also sent out and came back negative. IgG4 serological testing showed IgG4 levels of 187 mg/dL. Due to the patient's comorbidities and overall clinical condition, the multidisciplinary managing team, including thoracic surgery, hepatobiliary, and immunology specialists, opted against any intervention for the patient.

## Discussion

IgG4-RD is a multifaceted condition with the potential to impact a wide array of organ systems. Among these, the respiratory system, particularly the pleura, represents one of the less frequently affected areas. The case under discussion introduces a patient exhibiting a rare manifestation of IgG4-related respiratory disease, characterized by pleural effusion and a mass located in the right hemidiaphragm. The diverse involvement of multiple systems, occasionally concurrently, results in a highly variable clinical presentation for each individual. While autoimmune pancreatitis, Mikulicz’s disease (enlargement of salivary and lacrimal glands), orbital disease, tubulointerstitial nephritis, and retroperitoneal fibrosis are among the more prevalent presentations, pulmonary involvement is observed in 10-30% of cases, in contrast to pancreatic involvement, which is seen in 20-60% of cases [1]. The spectrum of pulmonary manifestations is broad, encompassing nodules, masses, ground-glass opacities, pleural effusion, interstitial lung disease, and more. Nodular lesions and bronchovascular involvement have been identified as the predominant pulmonary manifestations [5]. In the context of exudative pleural effusion, once malignancy is excluded, IgG4-RD should be contemplated, especially when other signs are present. This case underscores the significance of recognizing both a rare presentation of IgG4-RD and an infrequent cause of unexplained pleural effusion.

The diagnostic approach for IgG4-RD is multifaceted, necessitating histopathological validation in conjunction with laboratory tests, imaging studies, and clinical acumen. Typically, serum studies or serology will indicate an elevated serum IgG4 level, with sensitivity and specificity ranging from 83-97% and 60-85%, respectively. Approximately two-thirds of patients exhibit this elevation [1,6]. A serum IgG4 level exceeding 135 mg/dL is generally recognized as the diagnostic threshold for IgG4-RD [1,7]. Additional laboratory findings might encompass hypergammaglobulinemia and mild to moderate eosinophilia. Histopathological evaluation remains pivotal for both diagnosis and differentiation from conditions that mimic IgG4-RD. Three cardinal features are sought: IgG4-positive plasma cell-rich lymphoplasmacytic infiltrates, storiform fibrosis, and obliterative phlebitis [6]. However, it is imperative to acknowledge that storiform fibrosis might not be a dominant feature in pulmonary or thoracic IgG4-RD, unlike other organ systems, as evidenced by multiple studies [8]. The patient in this case predominantly exhibited IgG4-positive plasma cell-rich lymphoplasmacytic infiltrates, with an absence of storiform fibrosis and obliterative phlebitis. This case contributes to the growing body of evidence suggesting that thoracic histopathology in IgG4-RD might differ from other manifestations. Consequently, a holistic diagnostic approach, integrating laboratory values, histopathological findings, and clinical context, is paramount.

Similar cases of IgG4-related pleural effusion have been documented in the literature, underscoring the importance of accurately diagnosing this uncommon presentation [9-11]. In these other cases, effective clinical management was crucial due to the unusual etiology of the disease. These factors, combined with the reported instances, emphasize the significance of considering this rare disease in the differential diagnosis of unexplained pleural effusion.

A potential limitation of this case is the absence of comprehensive data detailing the management of this disease in our patient. Glucocorticoids remain the cornerstone of first-line treatment. However, given the patient's advanced age and multiple comorbidities, both medical and surgical interventions, especially concerning the hemidiaphragm mass, were deemed inappropriate, leading to supportive care.

## Conclusions

This report delineates the case of a 99-year-old male presenting with unexplained pleural effusion and an incidental mass in the right hemidiaphragm. Subsequent investigations, coupled with clinical suspicion, pointed towards a diagnosis of IgG4-RD. This case not only showcases a seldom-seen manifestation of IgG4-RD but also emphasizes the condition's vast array of presentations. Furthermore, it accentuates a potential etiology for unexplained exudative pleural effusion and underscores the potential variability in thoracic IgG4-RD histopathology. It is imperative for clinicians to maintain a heightened awareness of this condition. If IgG4-RD is not considered in the differential diagnosis, specific stains might not be ordered, potentially leading to missed diagnoses. This can result in the condition being underdiagnosed, underscoring the necessity for a high index of suspicion. Further research is warranted to elucidate the true prevalence and treatment outcomes concerning pleural IgG4-RD.
